# Ethylene production with engineered *Synechocystis* sp PCC 6803 strains

**DOI:** 10.1186/s12934-017-0645-5

**Published:** 2017-02-23

**Authors:** Vinod Puthan Veetil, S. Andreas Angermayr, Klaas J. Hellingwerf

**Affiliations:** 1 Molecular Microbial Physiology Group, Swammerdam Institute for Life Sciences, University of Amsterdam, Science Park 904, 1098 XH Amsterdam, The Netherlands; 2 Reliance Technology Group, Reliance Industries Ltd., Navi Mumbai, India; 3 Institute of Science and Technology (IST) Austria, Klosterneuburg, Austria

**Keywords:** Ethylene, Cyanobacteria, Sustainable, Synechocystis, Glycogen, Oxoglutarate, Arginine

## Abstract

**Background:**

Metabolic engineering and synthetic biology of cyanobacteria offer a promising sustainable alternative approach for fossil-based ethylene production, by using sunlight via oxygenic photosynthesis, to convert carbon dioxide directly into ethylene. Towards this, both well-studied cyanobacteria, i.e., *Synechocystis* sp PCC 6803 and *Synechococcus elongatus* PCC 7942, have been engineered to produce ethylene by introducing the ethylene-forming enzyme (Efe) from *Pseudomonas syringae* pv. *phaseolicola* PK2 (the Kudzu strain), which catalyzes the conversion of the ubiquitous tricarboxylic acid cycle intermediate 2-oxoglutarate into ethylene.

**Results:**

This study focuses on *Synechocystis* sp PCC 6803 and shows stable ethylene production through the integration of a codon-optimized version of the *efe* gene under control of the P*trc* promoter and the core Shine–Dalgarno sequence (5′-AGGAGG-3′) as the ribosome-binding site (RBS), at the *slr0168* neutral site. We have increased ethylene production twofold by RBS screening and further investigated improving ethylene production from a single gene copy of *efe*, using multiple tandem promoters and by putting our best construct on an RSF1010-based broad-host-self-replicating plasmid, which has a higher copy number than the genome. Moreover, to raise the intracellular amounts of the key Efe substrate, 2-oxoglutarate, from which ethylene is formed, we constructed a glycogen-synthesis knockout mutant (*ΔglgC*) and introduced the ethylene biosynthetic pathway in it. Under nitrogen limiting conditions, the glycogen knockout strain has increased intracellular 2-oxoglutarate levels; however, surprisingly, ethylene production was lower in this strain than in the wild-type background.

**Conclusion:**

Making use of different RBS sequences, production of ethylene ranging over a 20-fold difference has been achieved. However, a further increase of production through multiple tandem promoters and a broad-host plasmid was not achieved speculating that the transcription strength and the gene copy number are not the limiting factors in our system.

## Background

An increasing world population coupled with the need to reduce greenhouse gas emissions is driving the need to explore sustainable and renewable alternatives to the production of bulk fuels and chemicals from fossil sources. Ethylene is one of the most widely produced organic compounds and the raw material for polyethylene for consumables, ethylene oxide/glycol for polyethylene terephthalate (PET)’, resins for PET fiber, bottles and other packaging materials, and ethylene dichloride for PVC plastic uses in construction and piping. The global demand for the above products is leading to increased ethylene consumption which is expected to further grow at about 4% per year over the couple of years [17]. The traditional production methods based on steam cracking of naphtha from petroleum sources, and ethane from natural gas, are under pressure from society, due to the finite oil/gas supplies and large quantities of greenhouse gases produced (1.5–3.0 ton of carbon dioxide for every ton of ethylene produced). Hence there is an urgent need to explore fossil fuel free, sustainable, carbon–neutral ways to produce this important chemical and in this context, microbial biotechnology is leading the way.

Ethylene is biologically synthesized in plants and is an important plant hormone involved in regulation of biological processes like germination and fruit ripening [21, 37]. In higher plants, ethylene is produced from methionine in a three-step reaction via amino-propane carboxylic acid (ACC) in a pathway better known as the ACC pathway or the Yang cycle [1, 10]. Some microbes also produce ethylene, using two distinct pathways, neither of which is found in plants [10]. Most prokaryotes use methionine as the starting substrate and convert it into ethylene in a two-step process via 2-keto-4-methylthiobutyric acid (KMBA), catalyzed by an NADH:Fe-EDTA oxidoreductase (the KMBA pathway) [27]. Many plant pathogens, however, like *Pseudomonas syringae* and *Pencillium digitatum*, produce ethylene to weaken their hosts’ defense and they synthesize ethylene directly from 2-oxoglutarate, an important tricarboxylic acid (TCA) cycle intermediate, in an efficient single-step reaction, catalyzed by the ‘ethylene forming enzyme’ (Efe) [20, 26, 40]. This 2-oxoglutarate-dependent pathway has been expressed in a range of bacteria [9, 18, 19], fungi [6, 34] and yeast [28] and this has been covered in detail in a recent review [8].

Out of the various microbial model systems so far studied for heterologous expression of the Efe enzyme, metabolic engineering and synthetic biology of cyanobacteria [16, 31, 38], offers the most promising approach, as these photoautotrophic bacteria can use sunlight to perform oxygenic photosynthesis and thereby sequester and recycle carbon dioxide directly into ethylene. A techno-economic analysis of such a conceptual production process was also recently published [25]. The first studies on production of ethylene in cyanobacteria were carried out in *Synechococcus elongatus* PCC 7942 (hereafter referred to as *Synechococcus*), by introducing the *efe* gene from *Pseudomonas syringae pv*. *phaseolicola* PK2 (the Kudzu strain) with the promoter and terminator of the *efe* gene also from *P. syringae* [11] into this cyanobacterium. An ethylene production rate of 53 µl/l/h/OD_730_ was obtained. Replacing the native promoter of the *efe* gene with the promoter of the *psbAI* gene (from *Synechococcus*) and removing the terminator further increased the specific rate of ethylene formation sixfold to 323 µl/l/h/OD_730_ [30]. However, this strain was unstable due to the occurrence of homologous recombination events between the *psbAI* gene on the host’s chromosome and the *psbAI* and *efe* gene sequences on the plasmid, resulting in a rapid decrease in ethylene production in recombinant cyanobacterial cultures. To circumvent the above problem, the *efe* gene was then inserted into the chromosomal *psbAI* locus. This increased the rate of ethylene–formation to 451 µl/l/h/OD_730_, but the recombinant strain showed a lowered specific growth rate and was genetically unstable due to the presence of mutational hotspots [33]. Recently, stable and continuous ethylene production was achieved in *Synechocystis* sp. PCC 6803 (hereafter referred to as *Synechocystis*) in a study in which a codon-optimized *efe* gene (with mutational hot-spots eliminated) was expressed from a constitutive, pea plant chloroplast *psbA* promoter, and integrated at the *slr0168* neutral-site. A specific ethylene production rate of about 65 µL/L/h/OD_730_ was achieved in this way, in a strain with one copy of the *efe* gene, and grown with white light of an intensity of 50 μmol photons/m^2/^s^1^, and this production rate was more than doubled (i.e. to about 130 µL/L/h/OD_730_) in a strain with two copies of the *efe* gene. By further optimizing growth conditions, a peak volumetric production rate of 5600 µl/l/h and a continuous production rate of 3100 µl/l/h were achieved [35]. Stable ethylene production was also reported in *Synechocystis* by expressing the Kudzu Efe constitutively from a derivative of the broad-host range low-copy RSF1010 plasmid, under control of the P*trc* promoter, which resulted in production rates of about 170 µl/l/h/OD_730_ [14]. Very recently, high ethylene production rates were reported in a *Synechocystis* strain carrying multiple copies of the codon-optimized *efe* gene from *Pseudomonas syringae pv. sesame,* under control of the *PcpcB* promoter (selected after considerable promoter screening efforts) [41]. The authors further increased production rates by optimizing growth conditions and feeding the cells with 2-oxoglutarate, in combination with heterologous expression of a 2-oxoglutarate permease, to achieve an ethylene production rate of 858 µl/l/h/OD_730_.

In this study, we achieved stable ethylene production by integrating the codon-optimized *efe* gene into *Synechocystis* under control of the *Ptrc* promoter and the core Shine-Dalgarno sequence (5′-AGGAGG-3′) as the ribosome binding site (RBS), at the *slr0168* neutral site. We subsequently increased the ethylene production rate twofold through RBS screening. To better understand the bottlenecks towards further improving the rate of ethylene production, we studied gene expression using multiple consecutive promoters, and we used a broad host self-replicating plasmid, which has been reported to have about one to three times the copy number of the genome [4]. Moreover, instead of expressing a transporter and feeding the cells with 2-oxoglutarate, from which ethylene is formed by the Efe enzyme directly, we have engineered the cells to raise the intracellular generation of this key metabolite by knocking out the pathway towards glycogen synthesis and starving the cells for nitrogen.

## Methods

### Strains and growth conditions

*Escherichia coli* strains XL-1 Blue (Stratagene) or EPI400 (Epicentre Biotechnologies) were grown in lysogeny broth (LB) medium at 37 °C in a shaking incubator at 200 rpm or on plates augmented with 1.5% (w/v) agar. Antibiotics, kanamycin (50 µg/ml), chloramphenicol (35 µg/ml) and ampicillin (100 µg/ml) were added alone or in combination when needed.

*Synechocystis* sp. PCC 6803, obtained from D. Bhaya, University of Stanford, Stanford CA, was routinely cultivated in liquid BG-11 medium (Sigma-Aldrich), at 30º C, in a shaking incubator, under constant white light illumination (30 μmol photons/m^2^/s^1^). Cultures were supplemented with 20 mM TES-KOH, pH 8.0. Kanamycin resistant strains were grown in medium containing 50 µg/ml kanamycin. BG-11 plates were made with 1.5% (w/v) agar and additionally supplemented with 10 mM TES-KOH, pH 8.0, 0.3% (w/v) sodium thiosulphate and 50 µg/ml kanamycin for resistant strains. For nitrogen starvation experiments, nitrogen free BG-11 medium (BG-11No) was used and supplemented with 1 mM NaNO_3_. Cells were routinely stored at −80 °C in BG-11 medium supplemented with 5% (v/v) DMSO. Growth was monitored by following the optical density at 730 nm (OD_730_).

Natural transformation, in wild-type *Synechocystis*, as well as in the glycogen-synthesis knock-out strain, was performed as described previously [2, 3, 7]. Transformants were subjected to increasing concentrations of antibiotics to drive chromosome segregation. Conjugation of the pVZ derived plasmids from *E. coli* XL-1 to *Synechocystis* (both wild-type and the glycogen-synthesis knock-out strain) was carried out by tri-parental mating using *E. coli* J53 (pRP4) as helper strain as reported previously [32]. Kanamycin (50 µg/ml) was used to select for positive clones. Correct insertion of genes or plasmids was verified by colony PCR’s using Taq DNA polymerase (Thermo-Scientific).

### Codon optimization and gene synthesis

The gene sequence of the ethylene-forming enzyme from *Pseudomonas syringae* pv. *phaseolicola* (Genebank: AAD16440.1) was taken from the NCBI protein database. Codon optimization and removal of restriction sites in the gene to be used for further cloning procedures was done using the OPTIMIZER application [29] and the codon usage table described in [32]. The gene was synthesized by GenScript (NJ, USA) and delivered as pSEQ_*efe*.

### Plasmid construction

Integration plasmids: The *efe* gene was amplified from pSEQ-*efe* using efe-bb-fwd (contains the RBS-aggagg) and efe-bb-rev (Table 1), cut with EcoRI/SpeI (insert) and ligated into pSB1AC3_TT cut with EcoR1/Xba1 (vector) to obtain pSB1AC3_TT_*efe*. The rbs_efe-tt construct from above plasmid was cloned in line with the *Ptrc* promoter in plasmid pSEQ_trc [2] to get plasmid pSEQ-*Ptrc*_*efe*-tt. The whole *efe* expression cassette *Ptrc*_*efe*_tt was then subcloned into the pHKH001 integration plasmid [2]. For integration of plasmids to express *efe* under different RBS sequences, the *efe* gene was amplified with primers containing the RBS sequences (Table 1) and cloned back into the pHKH001 integration vectors. The 3xP*trc* and 5xP*trc* constructs were synthesized and directly inserted into pHKH001 by GenScript. The *efe* gene amplified with the Pbs-30-containing primer was then cloned behind the multiple promoter sequences to obtain the plasmids pHKH-3x*trc*_rbs30_*efe*-tt and pHKH-5x*trc*_rbs30_*efe*-tt.

**Table 1:** Cloning parts, plasmids, strains and primers used in this study

Cloning parts, plasmids, strains and primers	Description	Source/remark
**RBS** ^a^		
Rbs-O	AGGAGGACTAGC**ATG**	[2]
Rbs-34	ATTAAAGAGGAGAAAACTAGC**ATG**	[15]
Rbs-30	AAAGAGGAGAAAACTAGC**ATG**	[15]
Rbs-H	TAGTGGAGGTACTAGC**ATG**	RBS* from [15]
Rbs-C9	AAAGGAGGTGATAGC**ATG**	This study
Rbs-C10	AAAGGAGGTGATTAGC**ATG**	This study
Rbs-C11	AAAGGAGGTGATCTAGC**ATG**	This study
**Promoter**		
P*trc*	TTGACAATTAATCATCCGGCTCGTATAATGTGTGGAATTGTGAGCGGATAACAATTTCACAC	[2]
3 × P*trc*	TTGACAATTAATCATCCGGCTCGTATAATGTGTGGAATTGTGTTGACAATTAATCATCCGGCTCGTATAATGTGTGGAATTGTGTTGACAATTAATCATCCGGCTCGTATAATGTGTGGAATTGTGAGCGGATAACAATTTCACACA	This study
5 × P*trc*	TTGACAATTAATCATCCGGCTCGTATAATGTGTGGAATTGTGTTGACAATTAATCATCCGGCTCGTATAATGTGTGGAATTGTGTTGACAATTAATCATCCGGCTCGTATAATGTGTGGAATTGTGTTGACAATTAATCATCCGGCTCGTATAATGTGTGGAATTGTGTTGACAATTAATCATCCGGCTCGTATAATGTGTGGAATTGTGAGCGGATAACAATTTCACACA	This study
**Terminator**		
tt	Same as B0014 from http://www.partsregistry.org	[2]
**Plasmids**		
pHKH001	Integration vector disrupting *slr0168* in *Synechocystis* genome with kanamycin resistance cassette a selection marker	[2]
pHKH_BB	Bio-brick compatible derivative of pHKH001 carrying the *Ptrc* promoter and tt-transcription terminator	This study
pHKH_rbsO_*efe*	Integration vector carrying *efe* under *Ptrc* promoter and Rbs-O	This study
pHKH_rbs34_*efe*	Integration vector carrying *efe* under *Ptrc* promoter and Rbs-34	This study
pHKH_rbs30_*efe*	Integration vector carrying *efe* under *Ptrc* promoter and Rbs-30	This study
pHKH_rbsH_*efe*	Integration vector carrying *efe* under *Ptrc* promoter and Rbs-H	This study
pHKH_rbsc9_*efe*	Integration vector carrying *efe* under *Ptrc* promoter and Rbs-C9	This study
pHKH_rbsc10_*efe*	Integration vector carrying *efe* under *Ptrc* promoter and Rbs-C10	This study
pHKH_rbsc11_*efe*	Integration vector carrying *efe* under *Ptrc* promoter and Rbs-C11	This study
pHKH_3xP*trc*_*efe*	Integration vector carrying *efe* under 3x*Ptrc* promoter and Rbs-30	This study
pHKH_5xP*trc*_*efe*	Integration vector carrying *efe* under 5x *Ptrc* promoter and Rbs-30	This study
pVZ_LS	A bio-brick compatible version of mobilizable broad host self-replication plasmid pVZ321 containing the RSF1010 replicon, and carrying the *Ptrc* promoter, Rbs30 and tt-transcription terminator	Plasmid pVZ321 from [42]
pVZ-*efe*	pVZ plasmid carrying *efe* gene under *Ptrc* promoter and rbs 30	This study
pVZ_Nhis-*efe*	pVZ plasmid carrying N-terminal hexa-histidine tagged *efe* under promoter *Ptrc* and Rbs-30	This study
**Strains**		
Wild-type (WT)	*Synechocystis* sp. PCC6803, glucose tolerant, naturally transformable	Bhaya (Stanford)
VPV1	WT, Slr0168::*Ptrc*-rbsO-*efe*-Km^r^	This study
VPV2	WT, Slr0168::*Ptrc*-rbs34-*efe*-Km^r^	This study
VPV3	WT, Slr0168::*Ptrc*-rbs30-*efe*-Km^r^	This study
VPV4	WT, Slr0168::*Ptrc*-rbsH-*efe*-Km^r^	This study
VPV5	WT, Slr0168::*Ptrc*-rbsc9-*efe*-Km^r^	This study
VPV6	WT, Slr0168::*Ptrc*-rbsc10-*efe*-Km^r^	This study
VPV7	WT, Slr0168::*Ptrc*-rbsc11-*efe*-Km^r^	This study
VPV40	WT, Slr0168::3x*Ptrc*-rbsO-*efe*-Km^r^	This study
VPV43	WT, Slr0168::5x*Ptrc*-rbsO-*efe*-Km^r^	This study
SAW11	Glycogen knock out strain	[36]
VPV55	WT carrying plasmid pVZ _*efe*	This study
VPV56	SAW11 carrying plasmid pVZ _*efe*	This study
VPV58	SAW11 carrying plasmid pVZ_Nhis_*efe*	This study
VPV62	WT carrying plasmid pVZ_Nhis_*efe*	This study
VPV65	SAW11, Slr0168::*Ptrc*-rbs30-*efe*-Km^r^	This study
**Primers**		
efe-bb-fwd	GCGGAATTCGCGGCCGCTTCTAGAGGAGGACTAGCATGACCAACTTGCAAACCTTTGAAT	
efe-bb-r	GTACTGCAGCGGCCGCTACTAGTATTAGGAGCCGGTGGCGCG	
vpv-1-st-Hind-R	CTAGTAAAGCTTATCAATACTTTCCACCCC	
VPV-efe-Bamh1-f-noatg	CCATCACGGATCCACCAACTTGCAAACCTTTGAATTGC	
VPV-efe-st-Hind3-rev	TAATTAAGCTTATCAGGAGCCGGTGGCGCGGGTATCGG	
V-rbs30-efe-f	GACAGCTAGCATTAAAGAGGAGAAAACTAGCATGACCAACTTGCAAACC	
V-rbs34-efe-f	GACAGCTAGCAAAGAGGAGAAAACTAGCATGACCAACTTGCAAACC	
V-rbsC10-efe-f	GACAGCTAGCAAAGGAGGTGATTAGCATGACCAACTTGCAAACC	
V-rbsC11-efe-f	GACAGCTAGCAAAGGAGGTGATCTAGCATGACCAACTTGCAAACC	
V-rbsC9-efe-f	GACAGCTAGCAAAGGAGGTGATAGCATGACCAACTTGCAAACC	
V-rbsH-efe-f	GACAGCTAGCTAGTGGAGGTACTAGCATGACCAACTTGCAAACC	
v-bbs-pUC-r	CGGGATCCGATCCAATCTGCAGCGGCCGCTACTAGTA	
H1-SEG-VF	TGTCGCCGCTAAGTTAGACCGC	
H2-SEG-VR	CTGTGGGTAGTAAACTGGCAATGCC	
H1-SEQ-VF	CGGCAATGGTCCCAAAAT	
KanF-SEQ-VR	AGACGTTTCCCGTTGAAT	
pVZ321-SEQ-F	CGCAGGGCTTTATTGATT	
pVZ321-SEQ-R	CCCCCCCCACTCTATTGTA	
efe-n–H-f	CAGATATGACATATGCATCATCATCATCATCATACCAACTTGCAAACCTTTGAATTGC	
efe-ba-r	ACCTAGGTCAGTAGGATCCTTATTAGGAGCCGGTGGCGCGGGTATCG	

^a^ RBS sequence underlined and start codon ATG is shown in bold

Self-replicating plasmids: a bio-brick compatible pVZ321 derivative, already carrying the *Ptrc* promoter and Rbs-30, was used as the starting point. The *efe* gene was amplified, and inserted into the NdeI/BamHI site of the pVZ321 derivative to obtain plasmids pVZ-*efe.*

### Measurement of the biomass-specific rate of ethylene production

Ethylene production experiments were carried out in triplicate cultures that were in the late exponential growth phase prior to harvesting. Cultures were harvested around OD_730_ = 1 and were assayed. A 4 ml culture volume was transferred to a 20 ml crimp cap glass vial and 40 µl of 1 M sodium bicarbonate solution (resulting in 10 mM final concentration) was added. The vial was sealed with a butyl rubber stopper, and incubated at room temperature on a flat orbital shaker placed under a panel of fluorescent white lights, with a light intensity of about 250–300 μmol photons/m^2^/s^1^, for 2 h, after which the culture was heated to 80 °C for 10 min to stop ethylene production and all further metabolism. The quantitative determination of the amount of ethylene produced was carried out on an Agilent trace-ultra gas analyzer, by injecting 1 ml of headspace and using a standard program for ethylene analysis. An ethylene standard curve was made by injecting known volumes of pure ethylene into blank 20 ml crimp cap glass vials, carrying 4 ml of BG-11 medium and 40 µl of 1 M sodium bicarbonate solution.

For ethylene production under nitrogen limitation conditions 35 ml cultures of OD_730_ = 1.0 were collected, washed once with BG11No supplemented with 1 mM NaNO_3_ and 10 mM sodium bicarbonate, and re-suspended in the same medium, and incubated at low light intensity (30 μmol photons/m^2^/s^1^) at 30 °C. After 18 h, 4 mL of each culture was added to gas chromatography (GC) vials in duplicate, with 10 mM bicarbonate each, and incubated at low light (30 μmol photons/m^2/^s^1^) for 3 h and growth was stopped by heating at 80 °C. Low light was used in this case because of the sensitivity of the glycogen-synthesis knockout strain (i.e. *∆glgC* strain and its derivatives) to high light. The same was repeated at 21, 24, 40, 46 and 63 h to study the dynamics elicited by nitrogen limitation. All figures were made using Grafit [22].

### Method for measuring 2-oxoglutarate

2-oxoglutarate levels were measured as reported earlier [36]. Briefly, after measuring the ethylene in the headspace, the glass vials were de-capped and culture centrifuged for 10 min at 12,000 rpm and the supernatant subsequently filtered (Sartorius Stedim Biotech, Minisart SRP4, 0.45 μm). Separation of organic acids was achieved by application of a 20-μL aliquot on a Rezex ROA-Organic Acid H^+^ (8%) column (Phenomenex), coupled to a refractive index detector (Jasco, RI-1530), using a flow of 0.5 mL/min and a column temperature of 45 °C.

### Ethylene production in aerated batch culture

Ethylene production under aeration was tested with the best strain, i.e. VPV3. This was carried out in a 1 l flat bottle fitted with a gas dispersion tube with a bottom frit at room temperature. A late exponential seed culture was diluted into 1 l of BG-11 medium and an initial OD_730_ of around 0.5 and cultivated for the next 7 days. The culture was continuously bubbled with CO_2_:AIR = 1:100 and the flow was maintained around 100 ml/min, Light was gradually increased from 30 to 220 μmol photons/m^2^/s^1^. From the third day onwards, 4 ml culture was withdrawn (in triplicate) and transferred to a 20 ml glass vial and sealed and incubated under the same conditions as the flat bottle reactor for 1 h, after which the culture was heated to 80 °C for 10 min to stop ethylene production (and all further metabolism). The amount of ethylene produced was determined as reported above.

## Results

### Introduction of the *efe* gene under control of the P*trc* promoter in *Synechocystis*

**Fig. 1:**
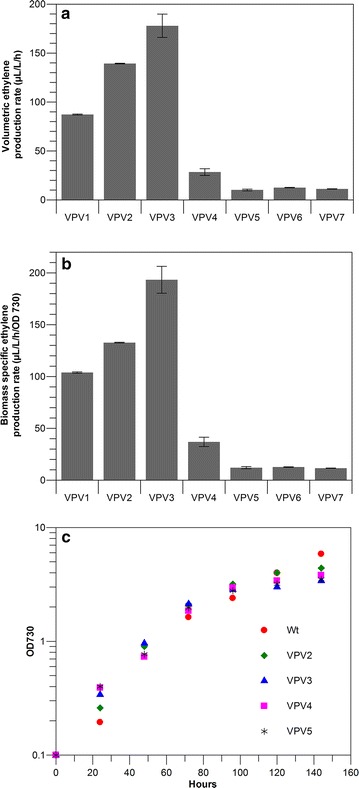
Rate of ethylene production in *Synechocystis* strains expressing the *efe* gene under control of the P*trc* promoter and various RBSs. **a** Comparison of volumetric ethylene production rates; **b** comparison of biomass specific ethylene production rates; **c** typical growth curves of the wild-type and ethylene producing *Synechocystis* strains. The genetic makeup of the VPV strains is detailed in Table 1. *Error bars* indicate standard deviation for triplicate measurements

The amino-acid sequence encoding the ethylene-forming enzyme from *Pseudomonas syringae* pv. *phaseolicola* (Genebank: AAD16440.1) was codon optimized for the preferred codon usage of *Synechocystis* and synthesized. The gene was introduced under the control of the P*trc* promoter and the ribosome binding site (RBS) AGGAGG (Rbs-O, which is the six-base consensus sequence found in case of 57% of *Escherichia coli genes* and 26% of *Synechocystis* genes contain the same [24], Table 1), in combination with the transcription terminator BB0014 (parts.igem.org) in the pHKH integration [2] plasmid using standard bio-brick assembly techniques. The final plasmid construct was confirmed by sequencing and transformed into *Synechocystis* by natural transformation for integration of the *efe* expression cassette and a kanamycin resistance cassette at the neutral docking site *slr0168*. Transformants were selected on kanamycin plates and segregated. Complete segregation was confirmed by colony PCR. Ethylene accumulation in the headspace of a closed culture vessel was quantified by gas chromatography with a flame ionization detector (GC-FID). The specific rate of rate of ethylene production observed for this strain (i.e. VPV1; see Table 1) was 103 ± 1 µl/l/h/OD_730_ (Fig. 1B).

### Optimization of the ribosome binding site

Using PCR amplification with primers with overhangs, we generated and subsequently cloned 6 RBS sequences: Rbs-34, Rbs-30, Rbs-H, Rbs-C9, Rbs-C10 and Rbs-C11 in front of the *efe* gene, replacing the initially used Rbs-O sequence. Rbs-34, Rbs-30, Rbs-H were reported in literature (BBa_B0034, BBa_B0030 and Rbs* respectively from [15]. The Rbs-C sequence is the exact complement to the 3′-end of the 16S RNA sequence of *Synechocystis*. Rbs-C9, Rbs-C10 and Rbs-C11 differed in the optimal aligned spacing (OAS) between the RBS and the start codon, as described in [24], to determine whether the OAS has an impact on the level of *efe* expression and hence ethylene production. Using the above constructs, integration plasmids were made and transformed into *Synechocystis*. Transformants were obtained and they were also subjected to the procedure for segregation between the wild type- and mutant genotype. Successful segregation was confirmed by colony PCR. The strains carrying Rbs-34 (VPV2), Rbs-30 (VPV3), Rbs-H (VPV4), and Rbs-C9 (VPV5), Rbs-C10 (VPV6) and Rbs-C11 (VPV7) were grown in batch culture, to compare their growth (rate) with that of the wild type (Fig. 1). No significant growth defects were observed for any of the constructs, indicating that overexpression of the Efe enzyme has no severe effect on growth (rate) under the measured conditions. GC analysis for ethylene detection was carried out as described in the Methods section. All strains carrying the *efe* gene produced ethylene, and the biomass specific rate of ethylene production was quantified (Fig. 1). The strains based on Rbs-C, which is exactly the complement of the consensus 3′-end of the 16S ribosome sequence of *Synechocystis*, produced the lowest amount of ethylene (Fig. 1. Moreover those strains (VPV5, VPV6 and VPV7) produced ethylene at similar rates indicating that the OAS does not have a strong impact on the expression of Efe. Strain VPV3 showed the maximum specific ethylene production rate, followed by VPV2 and VPV4. This is contrary to current literature, where Rbs-H (VPV4) is reported to be the RBS resulting in strongest expression amongst this set of sequences [15]. This may be explained by the notion that translation efficiency from the RBS is also dependent on its local sequence context, because of the secondary structure formed by the mRNA at the 3′ region, and hence is also dependent on the gene sequence around the RBS. The maximum rate of ethylene formation observed for strain VPV3 (i.e. with Rbs-30), was 195 ± 12 µl/l/h/OD_730_, which is double that of strain VPV1 (i.e. with Rbs-O). To validate if the differences in ethylene production rate can be indeed linked to the level of the Efe enzyme in the cells, cell free extracts were prepared and loaded on SDS-PAGE gel subsequently stained with Coomassie brilliant blue. However, no clear bands for the Efe protein could be detected in any of the strains tested (data not shown).

### Multiple promoters

**Fig. 2:**
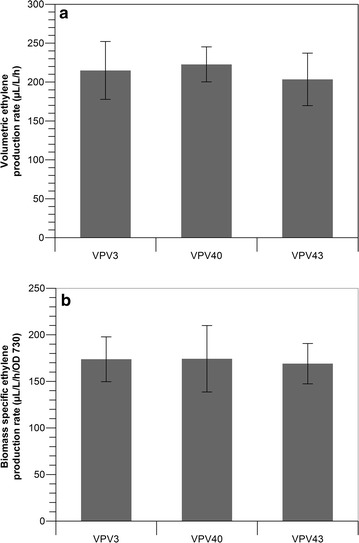
Rates of ethylene production form *Synechocystis* strains expressing the *efe* gene under control of in-tandem promoters. **a** Comparison of the volumetric ethylene production rate; **b** comparison of the biomass-specific rate of ethylene production. *Error bars* indicate standard deviation for triplicate measurements

Use of repeating identical promoters has been shown to increase expression of genes of interest in *E. coli* [23]. We have used a similar approach in *Synechocystis* and constructed two integration plasmids, one containing a triple P*trc* promoter (3×-P*trc*) and another one containing a quintuple P*trc* promoter (5×-P*trc*) in front of the Rbs-30-*efe* sequence, were made and confirmed by sequencing (Sequences see Table 1). The plasmids were transformed and the transformants were segregated as above, to obtain the strains VPV40 (3×-P*trc*) and VPV43 (5×-P*trc*). These strains were tested for their specific rate of ethylene production. Contrary to expectation, use of the in-tandem promoters did not lead to an increase in ethylene production (Fig. 2). Both strains VPV40 (3×-P*trc*) and VPV43 (5×-P*trc*) showed no significant difference in ethylene production compared to VPV3 (1×-P*trc*). This may suggest that these in-tandem promoter sequences do not increase expression significantly in our ethylene producing *Synechocystis* strains. Increased expression is expected to increase production as comparable ethylene producing systems [14, 35] show a corresponding increase in productivity. In those examples gene dosage has been increased through the introduction of two copies of the *efe* gene into the hosts’ genome.

### Comparison of an integrated *efe* construct with expression of *efe* from a plasmid

**Fig. 3:**
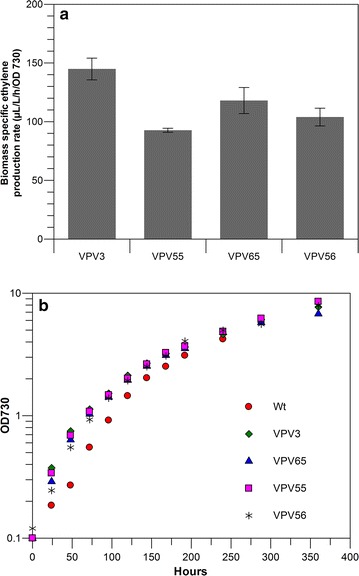
Comparison of rate of ethylene production between wild-type *Synechocystis*, expressing *efe* from a plasmid (VPV55) and as chromosomal integration (VPV3), and comparison with the corresponding *∆glgC* strains (VPV56 and VPV65). **a** Biomass specific production rates; **b** typical growth curves of ethylene producing *Synechocystis* strains mentioned above in comparison to their wild-type. *Error bars* in **a** indicate standard deviation for triplicate measurements

The broad host-range self-replicating plasmid pVZ321 (based on the RSF1010 replicon, [42] has been reported to be present in *Synechocystis* in numbers three times as high as the copy number of the genome, and has also been shown to express higher levels of protein as compared to the expression from an equivalent construct with chromosomal integration [3, 4]. So, inserting the *efe* expression cassette Ptrc-Rbs30-*efe* in this plasmid could lead to a higher level of Efe expression than in the equivalent strain with chromosomal insertion. The codon-optimized *efe* gene was inserted behind a *Ptrc* promoter and Rbs30 in a bio-brick compatible pVZ321 derivative, to obtain the plasmid pVZ-*efe*. The vector was transformed into *Synechocystis* by conjugation. Transformants were selected on kanamycin plates and uptake of the plasmid was confirmed by colony PCR, to obtain the strain VPV55 (containing pVZ-*efe*). The strain was tested for ethylene production and compared with the chromosomal integration strain VPV3 (Fig. 3a). Interestingly, the strain carrying the plasmid produced less ethylene than the strain with the chromosomally integrated *efe* gene. Comparison of growth curves of these strains does not show a significant difference in the growth rate nor in the growth yield between the strain with the integrated gene (i.e. VPV3) and the plasmid carrying strains VPV55 and VPV62 (Fig. 3b). We therefore interpret this to be a consequence of the modest control that the amount of Efe enzyme will have on the in vivo rate of ethylene formation when the *efe* gene is expressed from the strong *trc* promotor in combination with a strong RBS.

### Ethylene production in a *∆glgC* strain under standard growth conditions

It is reported in literature that the deletion of the *glgC* gene (*slr1176*, which encodes glucose-1-phosphate adenyl transferase) of the glycogen-synthesis pathway, leads to accumulation of 2-oxoglutarate under nitrogen-limiting growth conditions [5, 13]. As 2-oxoglutarate is the substrate for the Efe enzyme for the production of ethylene, improving the intracellular concentration of 2-oxoglutarate may improve the rate of ethylene production. A glycogen synthesis knock-out strain was made by replacing the *glgC* gene with a chloramphenicol resistance cassette (SAW011), as has been reported elsewhere [36]. This strain was transformed with the pHKH_Ptrc_Rbs30_*efe*, to insert the *efe* gene into the *slr0168* locus, as was earlier done for wild type *Synechocystis*. Transformants were selected on kanamycin and segregated. The segregated strain was confirmed by colony PCR to obtain strain VPV65 (∆*glgC*-*efe*). The plasmid pVZ-*efe* was also conjugated into SAW011 and selected on kanamycin to obtain the corresponding strain VPV56 (∆*glgC*-pVZ-*efe*). The strains were then tested under standard growth conditions for the rate of ethylene production (Fig. 3a). Here again, it is observed that the strains carrying *efe* on a plasmid produce less ethylene than the strain in which the *efe* gene is integrated into the genome. However, the difference between VPV65 and VPV56 is much less pronounced than between the respective wild-type background strains (VPV3 and VPV55). Interestingly, the glycogen synthesis knockout strains, carrying the *efe* gene on a plasmid, produce more than the corresponding wild-type background strain carrying the same plasmid, while their growth curves do not show any pronounced negative effect on growth rate nor growth yield (Fig. 3b).

### Ethylene production in a glycogen knock-out strain under nitrogen limitation

**Fig. 4:**
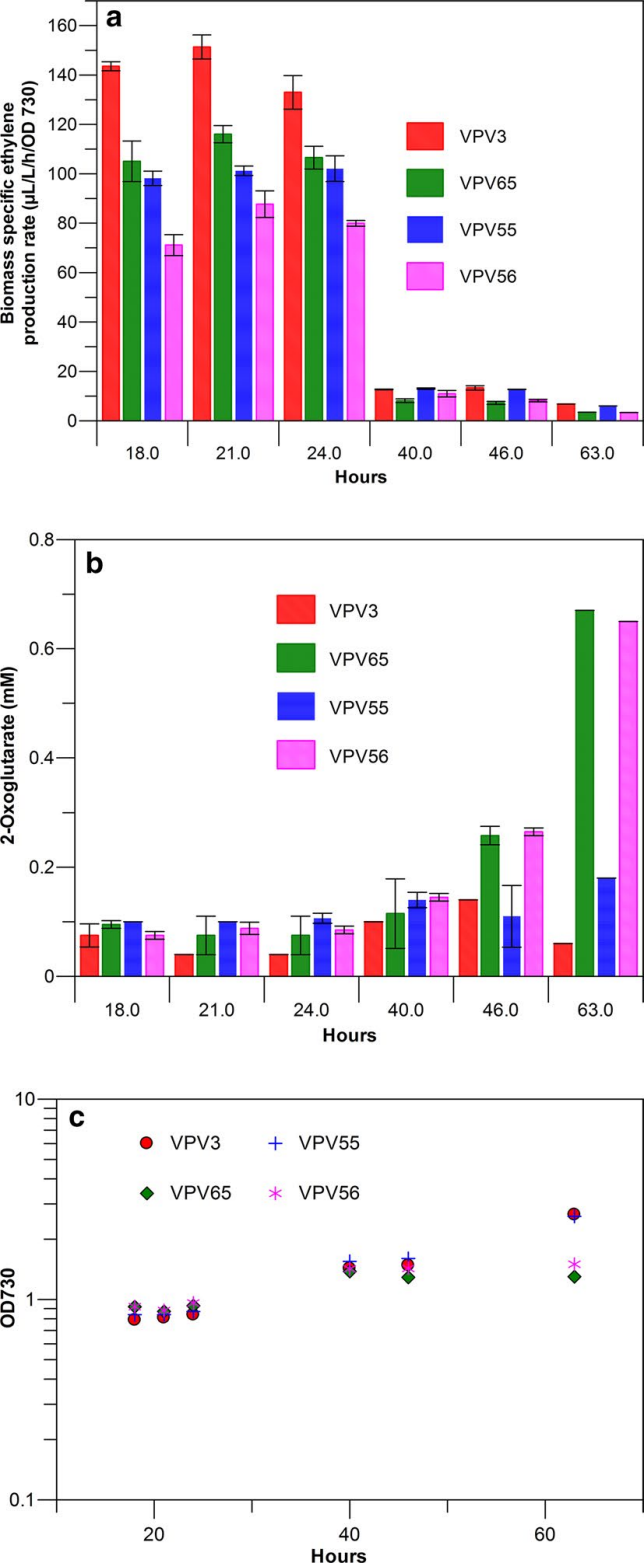
Comparison of ethylene production in wild type and in a *∆glgC* strain, with a plasmid- and a chromosome-incorporated *efe* gene. **a** Comparison of Biomass-specific ethylene production rates between wild-type *Synechocystis* strains expressing the *efe* gene from a plasmid (VPV55) or as a chromosomal integration construct (VPV3), in comparison to the glycogen-synthesis deficient *Synechocystis* strains expressing the *efe* gene on a plasmid (VPV56) or as a chromosomal integration construct (VPV65) under nitrogen limitation conditions, induced by growing the cells in nitrogen-restricted medium. **b** 2-Oxoglutarate measured in the supernatants of the ethylene-producing strains after measurement of the rate of ethylene-production. **c** Typical growth data of the mutant strains during the period in which the ethylene production experiments were conducted. *Error bars* indicate standard deviation for triplicate measurements for (**a**) and standard deviation for duplicate measurements for (**b**)

The ethylene producing wild-type background strains VPV3 (with an *efe* gene integrated on the genome) and VPV55 (with the *efe* gene on a plasmid) and the glycogen synthesis knock-out strains VPV65 (with an *efe* gene integrated on the genome) and VPV56 (with the *efe* gene on a plasmid) were selected for tests of ethylene production in nitrogen-limitation experiments. Exponential phase cultures were washed with nitrate free BG-11 medium (BG-11No), supplemented with 1 mM sodium nitrate, and then resuspended in the same medium. Samples were withdrawn after 18, 21, 24, 40, 46 and 63 h incubation at low incident light intensity (50 μmol photon/m^2/^s^1^) and ethylene production rates were determined in the same low-light conditions (Fig. 4). A clear drop in ethylene production is observed between 24 and 40 h even though the cultures show a positive change in OD_730_ indicating that (non-exponential) growth still continues. As expected, the wild type strain grows to an OD of about twice that of the glycogen synthesis deficient strains at 63 h, confirming observations reported in literature [13], on the ability of the wild-type strain to undergo a final division cycle under N-limitation. The glycogen synthesis knock-out strains VPV65 and VPV56, under nitrogen limitation, show higher 2-oxoglutarate levels than the wild-type background strain at the 46 and 63 h time points (Fig. 4b), and hence were expected to have produced more ethylene. However, at all time points, the wild-type background strains produce more than the glycogen synthesis knock-out strains, indicating that other factors such as a likely drop in the intracellular arginine concentration, or overall protein levels upon nitrogen starvation, now may be affecting the production rate of ethylene.

### Ethylene production in an aerated batch culture

**Fig. 5:**
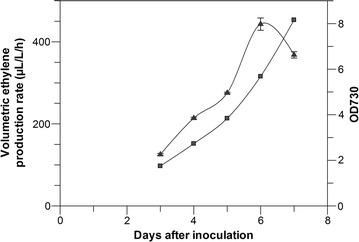
Ethylene production and growth with aerated batch cultivation. Volumetric ethylene production (*filled triangles*, levels indicated on the *left* y-axis) and OD_730_ (*filled triangles*, levels indicated on the *right* y-axis) plotted against time after inoculation. *Error bars* indicate standard deviation for triplicate measurements. *Lines* in the *figure* serve as a guide to the eye

The best ethylene-producing strain (VPV3, with an integrated *efe* gene and Rbs-30) was chosen for further production experiments. This strain was grown in a specially-designed 1 L flat glass bottle, fitted with a fine sparger, and grown for 7 days while the light intensity was gradually increased from 30 to 220 μmol photon/m^2^/s^1^. The volumetric ethylene production was then determined. We were able to achieve a maximum volumetric ethylene production rate of 443 µl/l/h at the higher light intensity of 220 μmol photon/m^2^/s^1^ (Fig. 5). This value is similar to the values reported by others [35] using a single copy of the *efe* gene but almost double the value reported for *efe* gene under the P*cpcB* promoter [41] indicating that the P*trc* promoter may be a stronger promoter than the P*cpcB* promoter. A similar observation is reported recently for isoprene production in *Synechococcus elongatus* [12].

## Discussion

Here we test several classical approaches from metabolic engineering with the aim to increase ethylene production in a genetically modified cyanobacterium. We systematically compare 7 different ribosomal binding sites with respect to ethylene production levels. We find a different ranking of the ribosomal binding sites compared to a previous expression study in *Synechocystis* which quantified heterologous expression level by GFP fluorescence using partially the same (overall 4) ribosomal binding sites [15]. Accordingly, we show that a broad range of productivities (20-fold difference) can be achieved through different RBS sequences, However, this variation is not a function of the RBS sequence alone; i.e. it is also dependent on the gene sequence following the RBS, and hence the way forward will be to further engineer ribosome binding sites to achieve higher productivities. Indeed, using an engineered ribosome binding site, [39] have recently shown 3.9-fold higher ethylene productivity in a *Synechocystis* strain carrying a single copy of efe gene per genome. Next we adopted the strategy of adding identical promoters repeatedly upstream the gene of interest, an approach which had resulted in successful subsequent GFP-overexpression in *E. coli* [23]. However, in our case a single copy of P*trc*, three and even five copies, all result in similar ethylene production rates. It has been suggested that a RSF1010-based plasmid, which is widely used in cyanobacterial research, reaches higher a copy-number per cell than the genome [4] and results in higher expression of a heterologous gene with subsequent higher rates of product formation [3]. Here, however, we do not find higher ethylene production. Thus the best-performing RBS in combination with subsequent transcription-increasing strategies (in-tandem promoters, plasmid-based expression) does not increase the productivity. These experiments are therefore most straightforwardly interpreted by assuming that the gene copy number is not the limiting factor in our system. Rather, the activity of the ethylene forming enzyme may have reached such a (high) level already so that the control by the amount of Efe activity over the flux towards the product is low or even absent. Therefore, we then asked if increased substrate availability would increase the rate of ethylene production: N-limitation in a glycogen-knockout strain results in intra- and extracellular accumulation of 2-oxoglutarate; however, also this did not result in higher ethylene production levels suggesting that the bottleneck is elsewhere in metabolism. A key factor could be the levels of arginine in the cells. The ethylene-forming enzyme uses arginine both as a cofactor as well as a substrate and this has a direct influence on the rate of ethylene production. Finally we use the best performing strain for ethylene production in 1 l batch cultures and achieved stable ethylene production for 1 week with maximum volumetric ethylene production rate of 443 µl/l/h at a light intensity of 220 μmol photon/m^2^/s^1^.
